# Comparative effectiveness and safety of glargine 300 U/mL versus degludec 100 U/mL in insulin-naïve patients with type 2 diabetes. A multicenter retrospective real-world study (RESTORE-2 NAIVE STUDY)

**DOI:** 10.1007/s00592-022-01925-9

**Published:** 2022-07-21

**Authors:** Gian Paolo Fadini, Raffaella Buzzetti, Antonio Nicolucci, Monica Larosa, Maria Chiara Rossi, Domenico Cucinotta, On behalf of the RESTORE-2 Study Group, Gabellieri Enrico, Marangoni Alberto, Pagotto Uberto, Bongiorno Claudio, Gatta Concetta, Del Buono Andrea, Lamacchia Olga, Maiellaro Pasquale, Antenucci Daniela, Brandoni Gabriele, Borroni Francesca, Gregori Giovanna, Di Benedetto Antonino, Placentino Giuseppe, Cavalot Franco, Barale Cristina, Fadini Gian Paolo, Del Sindaco Paola, Di Loreto Chiara, Anichini Roberto, Citro Giuseppe, D’Angelo Paola, Carletti Silvia, Buzzetti Raffaella, Sterpetti Sara, Carmen Mignogna, Elli Paolo

**Affiliations:** 1 Department of Medicine, University of Padova, Padua, Italy; 2 Department of Experimental Medicine, Sapienza University, Rome, Italy; 3 CORESEARCH – Center for Outcomes Research and Clinical Epidemiology, Pescara, Italy; 4 Medical Affairs, Sanofi, Milan Italy; 5 Department of Clinical and Experimental Medicine, University of Messina, Messina, Italy

**Keywords:** Type 2 diabetes, Basal insulin, Naïve, Glargine 300, Degludec 100, Effectiveness, Safety

## Abstract

**Aims:**

This study assessed comparative effectiveness of glargine 300 U/mL (Gla-300) versus degludec 100 U/mL (Deg-100) in insulin-naïve patients with T2D.

**Methods:**

This is a retrospective, multicenter, non-inferiority study based on electronic medical records. All patients initiating Gla-300 or Deg-100 were 1:1 propensity score-matched (PSM). Linear mixed models were used to assess the changes in continuous endpoints. Incidence rates (IR) of hypoglycemia were compared using Poisson’s regression models.

**Results:**

Nineteen centers provided data on 357 patients in each PSM cohort. HbA1c after 6 months (primary endpoint) decreased by − 1.70% (95%CI − 1.90; − 1.50) in Gla-300 group and − 169% (95%CI − 1.89; − 1.49) in Deg-100 group, confirming non-inferiority of Gla-300 versus Deg-100. Fasting blood glucose (BG) decreased by ~60 mg/dl in both groups; body weight remained unchanged. In both groups, the mean starting dose was 12U (0.15U/kg) and it was slightly titrated to 16U (0.20U/kg). IR (episodes per patient-months) of BG ≤70 mg/dl was 0.13 in Gla-300 group and 0.14 in Deg-100 group (*p*=0.87). IR of BG <54 mg/dL was 0.02 in both groups (*p*=0.49). No severe hypoglycemia occurred.

**Conclusion:**

Initiating Gla-300 or Deg-100 was associated with similar improvements in glycemic control, no weight gain and low hypoglycemia rates, without severe episodes during 6 months of treatment.

## Introduction

Improving glycemic control in type 2 diabetes (T2D) is a major goal of care, to reduce the incidence of micro- and macro-vascular complications [1, 2], but it is also a major challenge for patients, physicians and healthcare systems. In fact, inertia in intensifying diabetes therapy and especially in initiating basal insulin in patients with poor metabolic control is a well-recognized problem [3]. Many barriers exist and refer to clinicians, patients and settings; fear of hypoglycemia represents the main limitation to a prompt intensification [4, 5].

Second-generation basal insulin analogues (2BI) represent an opportunity to overcome these barriers. According to randomized controlled trials (RCTs), 2BI are non-inferior to first-generation basal insulins (1BI) with regard to the reduction of HbA_1c_ but safer in terms of hypoglycemia and with lower variability [6].

Two 2BI are currently available: insulin glargine 300 units/mL (Gla-300), commercialized in Italy in 2017, and degludec 100 units/ml (Deg-100), available since 2014.

Both insulins display a more stable pharmacokinetic and pharmacodynamic profile and a longer duration of action compared to the first-generation basal insulin glargine 100 units/mL (Gla-100) [6–10].

In the phase-3 studies from the EDITION clinical registration program [11–14], Gla-300 proved to be non-inferior to Gla-100 in T1D and T2D patients as well as among insulin naïve patients or switchers. A significantly lower percentage of patients with T2D or T1D experienced confirmed and/or severe nocturnal hypoglycemic events while being on Gla-300 compared to Gla-100 [11–14].

The BEGIN clinical registration program [15–18] showed comparable glycemic control of Deg-100 vs. Gla-100 and a lower rate of overall hypoglycemia in T2D and nocturnal hypoglycemia in T1D.

The randomized, head-to-head, parallel group BRIGHT study [19] involving insulin-naïve patients with T2D demonstrated that Gla-300 and Deg-100 provided similar glycemic control improvements with relatively low hypoglycemia risk. Hypoglycemia rate was comparable with the two basal insulins during the full study period, but lower with Gla-300 during the dose titration period. The CONCLUDE trial, involving people with T2D switching from 1BIs to Gla-300 or the new 200 U/mL formulation of insulin degludec, documented a similar rate of overall symptomatic hypoglycemia during the 36-week maintenance period [20].

Real-world evidence (RWE) is needed to assess effectiveness and safety of 2BI when prescribed in different settings [21], as the three real-world studies currently available [22–24], all conducted in US settings, provided controversial results. Therefore, real-world data on 2BI in patients with T2D are relatively scant, and most importantly, data from European countries are missing.

Given these premises, the RESTORE-2 study aimed at assessing the comparative effectiveness and safety of Gla-300 versus Deg-100 in a cohort of insulin-naïve patients with T2D followed under routine care in Italian diabetes outpatient clinics.

## Methods

### Study design and patients

The RESTORE-2 study was a retrospective, comparative, multicenter study. Inclusion criteria were: male or female gender, age ≥18 years, diagnosis of T2D (any disease duration), initiation of Gla-300 or Deg-100 from January 2017 to January 2020 and no previous treatment with basal insulin (naïve cohort) as recorded in the electronic medical records (EMRs). Exclusion criteria were: diagnosis of T1D, more than one type of basal insulin prescribed at index date or prescription of another basal insulin analogue in the six months after initiating insulin Gla-300 or Deg-100.

Anonymous patient data were derived from the same electronic chart system adopted at all participating centers (Smart Digital Clinic software—property of METEDA s.r.l.)

Centers recorded data on EMRs according to their clinical practice, taking into consideration that patients with T2D who need intensification are generally seen by the diabetologist on a 3-6 months basis. The date of the first prescription of the 2BI was considered as the index date (T0, baseline). All data relative to the period before and after the index date (± 6 months) for each patient were extracted and analyzed. Data recorded in the 6 months previous T0 were used to identify the baseline characteristics of patients, whereas data collected after 6 months (T6) represented the follow-up data. When more values of the same parameters were available during the before and after T0 periods, those recorded in the nearest date to T0 and T6 were considered. Data relative to the efficacy endpoints were considered only if values were recorded at T0±30 days and T6±30 days. The following characteristics were considered to describe the baseline patient profile: age, gender, diabetes duration, HbA1c, fasting blood glucose (FBG), weight/body mass index (BMI), total, basal and short-acting insulin dose, number of insulin injections, glucose-lowering drugs other than insulin, blood pressure, lipid profile, diabetes complications (low glomerular filtration rate, albuminuria, cardiovascular complications—i.e., myocardial infarction, coronary revascularization, coronary artery bypass, stroke, lower limb complications, peripheral artery disease—by ICD-9 CM codes).

Efficacy endpoints were: changes at 6 months (T6) in HbA_1c_, insulin doses, FBG and body weight (continuous endpoints); and frequency and proportion of patients with HbA1c <7% and HbA1c <8% at T0 and T6 (categorical endpoints). Changes in HbA_1c_ at 6 months from the Gla-300 or Deg-100 initiation represented the primary endpoint.

Safety endpoints were: episodes of hypoglycemia ≤70 mg/dl or <54 mg/dl from self-monitoring blood glucose tests (SMBG) downloaded in EMR (cutoffs recommended by ADA guidelines 2017) during 6 months, severe hypoglycemia (defined as “need of assistance by a third party” and reported in a dedicated module of the EMR) during 6 months.

In addition, the change in HbA_1c_ at 12 months was evaluated as a post hoc analysis in the subgroup of patients with available data.

### Statistical analysis

Sample size estimation was based on the primary endpoint, represented by the change in HbA1c levels after 6 months from the initiation of Gla-300 or Deg-100. We calculated that 296 patients per group were needed in order to achieve 80% power to detect non-inferiority using a one-sided, two-sample t-test. The margin of non-inferiority was set at 0.3%, which is generally considered a clinically meaningful difference. The true difference between the means was assumed to be 0.0. The significance level (alpha) of the test was 0.025. The standard deviation of HbA1c was assumed to be 1.3 in both groups.

Baseline patient characteristics according to the initiation of Gla-300 or Deg-100 were compared using the unpaired t-test or the Mann–Whitney U-test in case of normal and skewed continuous variables, respectively, and the Chi-square test or the Fisher exact test for categorical variables, as appropriate.

To allow for an unbiased comparison between patients initiating Gla-300 vs. Deg-100, a propensity score (PS) matching algorithm on a one-to-one basis was applied. To compute PS, we performed a logistic regression model taking into consideration age, gender, diabetes duration, baseline HbA1c, FBG, BMI, basal insulin dose, glucose-lowering drugs other than insulin as covariates. Variables included in the logistic model were those showing a statistically significant between-group difference at baseline [25]. A five-to-one greedy matching algorithm was used to identify a unique matched control in the initial Deg-100 group for each Gla-300 patient according to the individual PS. Adequacy of balance for the covariates in the matched sample was assessed via standardized mean difference between the 2 groups, considering differences less than 10% (absolute value) as indicative of a good balance.

PS matching was performed separately in the efficacy population and in the safety population.

Changes in HbA1c, FBG, body weight and insulin dose were assessed using mixed models for repeated measurements. Results are expressed as estimated mean or estimated mean difference from T0 with their 95% confidence interval (95% CI). Paired and unpaired t-test derived from linear mixed models for repeated measurements were applied for within-group and between-group comparisons, respectively.

As secondary outcomes, the proportions of patients with HbA1c <7.0% and HbA1c <8.0% at T0 and T6 were evaluated. Both within-group (McNemar test for change vs. baseline) and between-group (Chi-square test) statistical comparisons were applied.

Incidence rates (IR) of hypoglycemic events were calculated and expressed as numbers of events per patient-month with their 95% CI. Incidence of hypoglycemic events was compared between groups using Poisson’s regression model with correction for overdispersion.

The main analysis was conducted on the intention-to-treat (ITT) population, including all patients meeting eligibility criteria. The post hoc population included the subgroup of PS matched patients with a HbA1c value available at baseline and after 12 months.

For the evaluation of severe hypoglycemia, the safety population was represented by the ITT post-PS matching population (data derived from EMRs). For the evaluation of glycemic values ≤70 mg/dl and <54 mg/dl, a subsample of the safety population (PS matched patients having at least 1 SMBG value available) was considered.

## Results

### Patient disposition and characteristics

**Fig. 1:**
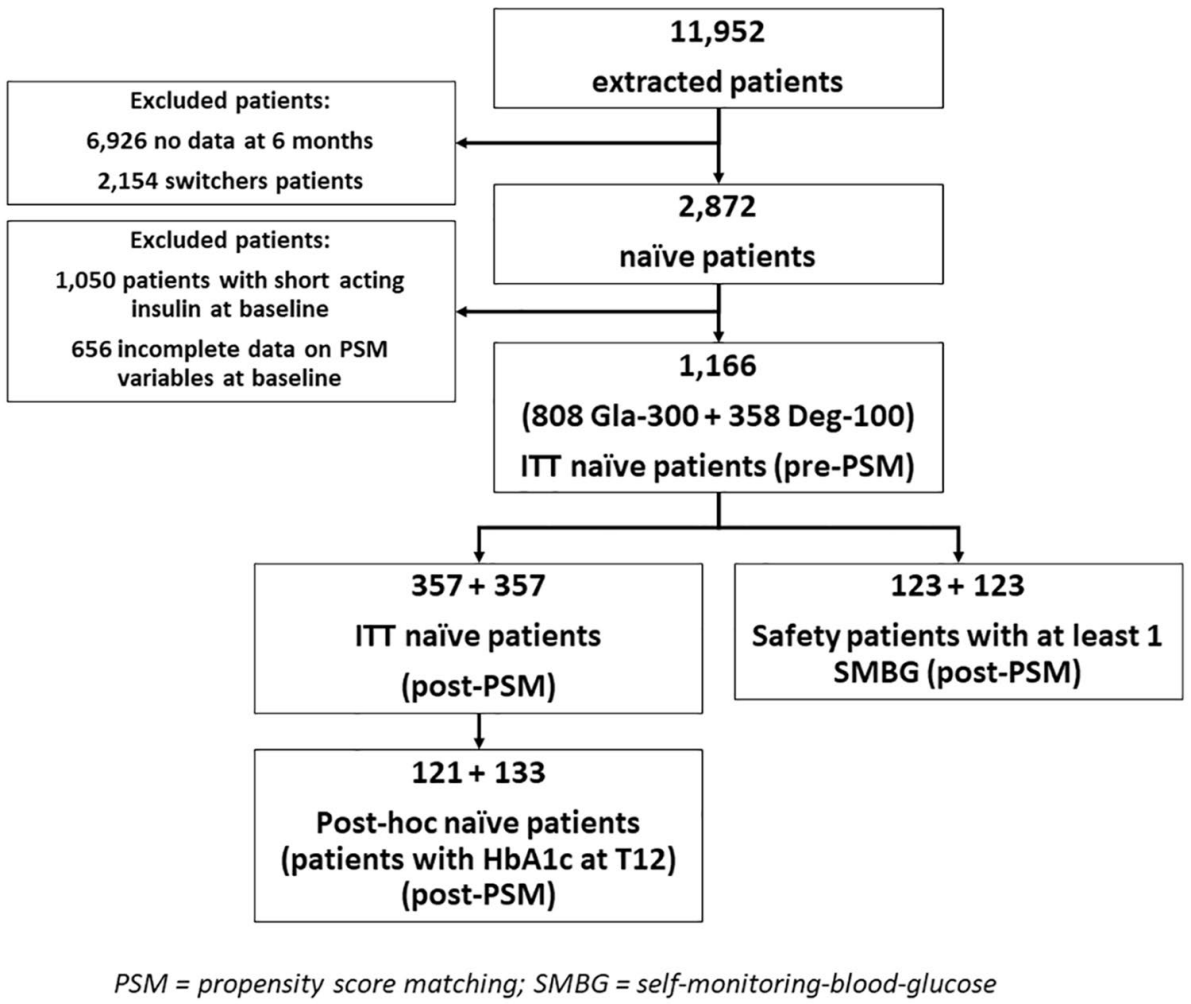
Study flowchart

Data were extracted from 19 centers (Fig. 1), yielding initial information from 1,166 patients (808 initiating Gla-300 and 358 initiating Deg-100), who were eligible insulin-naïve patients with first prescription of either 2BI as recorded in EMRs from January 2017 to January 2020. Centers with SMBG tests downloaded in the EMRs were 14 out of 19 (73.7%).

Before PS matching, age, gender, diabetes duration, FBG, HbA_1c_ were balanced between groups. On the other side, patients initiating Gla-300 (*N*=808) differed from those initiating Deg-100 (*N*=358) in terms of use of secretagogues (42.6% vs. 50.0%; *p*=0.02), glitazones (5.9% vs. 11.2%; *p*=0.002) and SGLT2i (27.5% vs. 19.6%; *p*=0.004) (Table 1). Differences of borderline statistical significance in BMI and use of GLP1-RA were also documented.

**Table 1:** Baseline patients’ characteristics—pre- and post-PSM ITT population

Variable	Pre-PSM			Post-PSM		
	Gla-300	Deg-100	*p*-value	Gla-300	Deg-100	*p*-value
N. Group	808	358		357	357	
Age (years)	68.7±12.0	69.8±10.9	0.26	68.7±11.7	69.8±10.9	0.32
Males (%)	59.9	59.8	0.97	60.8	59.7	0.76
Diabetes duration (years)	13.8±11.4	13.5±7.4	0.42	13.6±10.6	13.5±7.4	0.32
Weight (kg)	80.8±17.9	82.2±17.9	0.21	82.5±18.3	82.1±17.9	0.85
BMI (Kg/m2)	29.3±5.8	29.9±5.6	0.07	29.9±6.1	29.9±5.6	0.75
HbA1c (%)	9.3±1.8	9.2±1.6	0.26	9.3±1.9	9.2±1.6	0.87
HbA1c (mmol/l)	78.6±20.5	76.8±17.8	0.34	77.7±21.0	76.9±17.8	0.99
Fasting blood glucose (mg/dl)	210.1±70.0	202.0±63.0	0.13	212.3±74.9	201.9±63.1	0.16
Systolic blood pressure (mmHg)	135.2±19.1	135.2±16.5	0.76	135.6±19.0	135.1±16.5	0.81
Diastolic blood pressure (mmHg)	78.5±10.3	78.9±9.9	0.83	78.4±9.2	78.9±9.9	0.94
Total cholesterol (mg/dl)	178.8±44.7	178.0±41.7	0.96	178.7±43.0	178.1±41.7	0.86
LDL-cholesterol (mg/dl)	95.6±35.5	96.1±35.3	0.79	95.5±36.9	96.2±35.4	0.74
HDL-cholesterol (mg/dl)	46.7±13.5	46.0±12.0	0.85	46.6±12.9	46.0±12.1	0.84
Triglycerides (mg/dl)	189.8±182.0	187.7±150.6	0.83	186.9±172.8	187.9±150.8	0.86
eGFR <60 ml/min*1.73m^2^	34.4	32.8	0.73	34.1	32.8	0.81
Microalbuminuria (%)	32.8	31.0	0.68	31.5	31.0	0.93
Diabetes complications (%)	7.2	6.4	0.64	6.4	6.4	1.00
*Glucose-lowering therapy*						
Daily basal insulin dose (U)	11.6±4.8	12.4±6.3	0.21	11.8±5.5	12.5±6.3	0.24
*No. of glucose-lowering drugs other than insulin*						
<2	18.9	19.0	0.98	20.2	19.0	0.71
>=2	81.1	81.0		79.8	81.0	
Metformin (%)	80.0	79.9	0.98	77.0	79.8	0.36
Secretagogues (%)	42.6	50.0	**0.02**	45.1	49.9	0.20
Glitazones (%)	5.9	11.2	**0.002**	9.0	10.9	0.38
Acarbose (%)	4.3	4.7	0.75	3.1	4.8	0.25
DPPIV inhibitors (%)	49.9	49.2	0.82	50.4	49.0	0.71
GLP1-RAs (%)	18.6	23.5	0.054	22.1	23.5	0.66
SGLT2 inhibitors (%)	27.5	19.6	**0.004**	18.8	19.6	0.78

Data are means and standard deviations or frequencies and proportions. Variables included in the PSM: BMI and use of GLP1-RAs, SGLT2 inhibitors, glitazones and secretagogues.

*p*-values derived from unpaired t-test or the Mann–Whitney U-test in case of continuous variables and the Chi-square test or Fisher exact test for categorical variables, as appropriate. Statistically significant *p*-values (*p*<0.05) are in bold.

After PS matching, 357 subjects were included in each group (post-PSM ITT). The standardized differences of the variables before and after the PS matching are shown in Appendix 1. All selected variables had an absolute standardized mean difference >10% before matching and <10% after matching. The complete baseline characteristics of the ITT matched population are reported in Table 2.

**Table 2:** Changes in estimated mean levels of continuous endpoints during the follow-up by treatment and within-group and between-group comparisons (T3 vs. T0 and T6 vs. T0) (post-PSM ITT population)

Change in	Visit	Gla-300			Deg-100			Gla-300 vs. Deg-100	
		Estimated mean and 95% CI	Estimated mean difference from T0 and 95% CI	Within-group *p*-value*	Estimated mean and 95% CI	Estimated mean difference from T0 and 95% CI	Within-group *p*-value*	Between-groups difference (estimated mean and 95% CI)	Between-group *p*-value**
HbA1c (%)	T0	9.25 (9.07;9.43)	–	–	9.18 (9.00;9.36)	–	–	–	–
	T6	7.55 (7.42;7.68)	− 1.70 (− 1.90;− 1.50)	**<0.0001**	7.48 (7.35;7.61)	− 1.69 (− 1.89;− 1.49)	**<0.0001**	− 0.01 (− 0.29;0.27)	0.49
FBG (mg/dl)	T0	212.60 (204.86;220.34)	–	–	201.61 (193.73;209.49)	–	–	–	−
	T6	149.36 (144.07;154.65)	− 63.23 (− 71.95;− 54.51)	**<0.0001**	140.47 (135.00;145.94)	− 61.14 (− 70.06;− 52.22)	**<0.0001**	− 2.09 (− 14.56;10.38)	0.74
Body weight (Kg)	T0	82.55 (80.68;84.42)	–	–	82.12 (80.25;83.99)	–	–	–	–
	T6	82.28 (80.44;84.12)	− 0.26 − 0.72;0.20)	0.27	82.09 (80.25;83.93)	− 0.03 (− 0.49;0.43)	0.91	− 0.23 (− 0.88;0.42)	0.48
Daily basal insulin dose (U)	T0	11.79 (11.18;12.40)	–	–	12.45 (11.84;13.06)	–	–	–	**− **
	T6	16.25 (15.28;17.22)	4.45 (3.63;5.27)	**<0.0001**	15.99 (15.03;16.95)	3.54 (2.73;4.35)	**<0.0001**	0.92 (− 0.23;2.07)	0.12
Daily basal insulin dose (U/Kg)	T0	0.15 (0.14;0.16)	–	–	0.16 (0.15;0.17)	–	–	–	–
	T6	0.20 (0.19;0.21)	0.05 (0.04;0.06)	**<0.0001**	0.20 (0.19;0.21)	0.04 (0.03;0.05)	**<0.0001**	0.01 (–0.01;0.03)	0.06

Statistically significant *p*-values (p<0.05) are in bold

^*^Paired t-test derived from linear mixed models for repeated measurement

^**^Unpaired t-test derived from linear mixed models for repeated measurements

### Comparative effectiveness analysis

The mean follow-up time of patients was similar in the Gla-300 and Deg-100 groups (6.4±1.4 vs. 6.6±1.4 months, *p*=0.17), as was the mean number of visits per patient during 6 months (3.0±1.3 vs. 3.0±1.2, *p*=0.79).

**Fig. 2:**
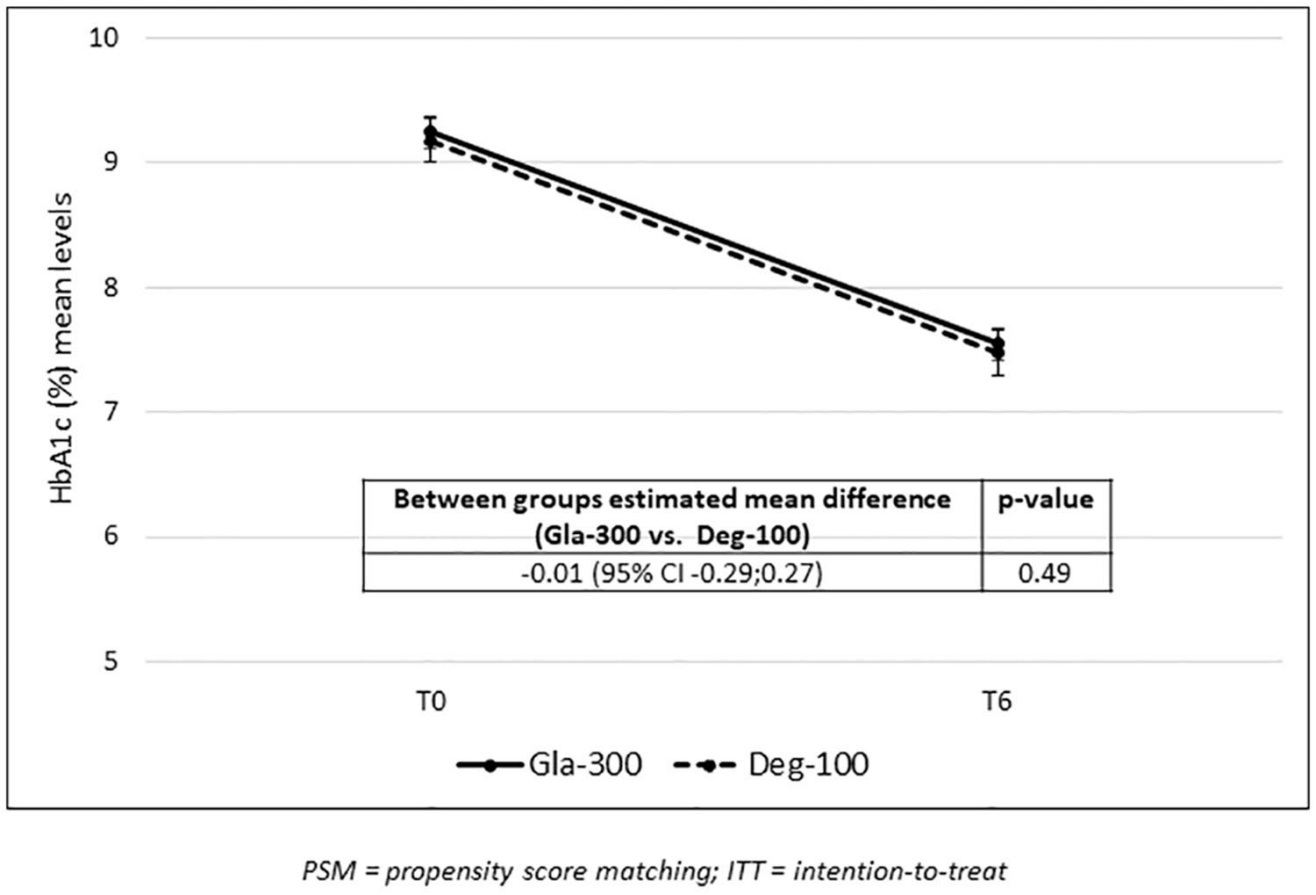
Changes in HbA1c estimated mean levels during the follow-up by cohort (post-PSM ITT population)

Both groups had estimated mean levels of HbA_1c_ at baseline of about 9.2%. For Gla-300 group, a statistically significant reduction in HbA_1c_ levels from baseline to 6 months (− 1.70%, 95% CI − 1.90; − 1.50) was documented. Similarly, for Deg-100 group, a statistically significant reduction in HbA_1c_ levels from baseline to 6 months (− 1.69%, 95% CI − 1.89; − 1.49) was documented. No between-group difference emerged in the change from baseline in HbA_1c_ (− 0.01%, 95% CI − 0.29; 0.27; *p*=0.49) (Table 2 and Fig. 2). The non-inferiority of Gla-300 vs. Deg-100 was confirmed (margin of non-inferiority of 0.30%; actual upper 95% CI at 6 months 0.27%).

In the Gla-300 group, the proportion of patients achieving HbA_1c_ levels <7.0% increased from 6.2% at T0 to 29.1% at T6 (*p*<0.0001), whereas in Deg-100 group, it increased from 4.8% at T0 to 32.9% at T6 (*p*<0.0001), with no between-group difference (p=0.31) (Appendix 2). In the Gla-300 group, the proportion of patients achieving HbA_1c_ levels <8.0% increased from 21.6% at T0 to 69.5% at T6 (*p*<0.0001), whereas in Deg-100 group it increased from 24.9% at T0 to 71.9% at T6 (*p*<0.0001), with no between-group difference (*p*=0.52) (Appendix 2).

Results of longitudinal models relative to secondary continuous endpoints are shown in Table 2.

At baseline, mean levels of FBG were 212.6 mg/dl in Gla-300 group and 201.6 mg/dl in Deg-100 group. In both groups, statistically significant reductions from baseline to 6 months were shown: − 63.23 mg/dl in Gla-300 group and − 61.14 mg/dl in Deg-100 group, with no between-group differences (*p*=0.74) (Table 2).

Not significant changes in body weight were documented in both groups after 6 months (Table 2).

Mean basal insulin dose was titrated during 6 months and statistically significant within-group increases were observed at T6 in both groups (Table 2). In the Gla-300 group, the estimated mean starting dose (T0) was 11.79 U and increased on average by +4.45 U at T6; in Deg-100 group, the estimated mean starting dose (T0) was 12.45 U and increased by +3.54 U at T6. In both groups, per-kg basal insulin dose significantly increased during the follow-up; at T6, the dose was of 0.20 U/kg in both groups. No between-group differences were found in insulin dose changes over time.

At the post hoc analysis, in the subgroup of patients with a HbA_1c_ value at 12 months (121 in Gla-300 group and 133 in Deg-100 group), a marked reduction in HbA_1c_ mean levels was maintained in both groups; the reduction was − 1.71% in Gla-300 versus − 1.44% in Deg-100 group, although the between-group difference did not reach the statistical significance (*p*=0.052) (Appendix 5).

### Comparative safety analysis

The safety population (i.e., ITT patients with at least 1 SMBG downloaded in the EMRs) was PS matched for the following unbalanced variables at T0: diabetes duration, HbA_1c_, number of glucose-lowering drugs other than insulin (<2 or >=2), use of metformin and secretagogues. Each PS matched group included 123 subjects (Appendix 3 and 4). No difference in the incidence of episodes of BG≤70 mg/dl or <54 mg/dl was present before insulin initiation in the two groups (Appendix 6).

Overall, 18,353 SMBG tests were available in Gla-300 group, and 19,621 SMBG tests were available for Deg-100 group. The incidence of BG events ≤70 mg/dl and <54 mg/dl during 6-month follow-up was very low and similar in Gla-300 group and in Deg-100 group. No between-group differences emerged (Table 3).

**Table 3:** Incidence rate of hypoglycemic events (BG ≤70 mg/dl and <54 mg/dl) during the 6-month follow-up by treatment and between-group difference (Safety population subsample: post PSM ITT patients having at least 1 SMBG value available)

Outcome	Group	Subjects	N SMBG	Events	Person-months	IR (95%CI)	IRR (95%CI)	Between-group *p*-value
BG ≤70 mg/dl	Gla-300	123	18,353	81	615	0.13 (0.07;0.26)	0.92 (0.36;2.38)	0.87
BG ≤70 mg/dl	Deg-100	123	19,621	90	631.7	0.14 (0.07;0.27)		.
BG < 54 mg/dl	Gla-300	123	18,353	15	615	0.02 (0.01;0.05)	1.54 (0.45;5.30)	0.49
BG < 54 mg/dl	Deg-100	123	19,621	10	631.7	0.02 (0.01;0.04)		.

No severe hypoglycemic episodes (evaluated in the safety population) were reported on EMRs in both groups.

## Conclusions

In this real-world study, comparative effectiveness analyses showed that initiating Gla-300 or Deg-100 in uncontrolled insulin-naïve patients with T2D was followed by statistically significant and clinically relevant HbA_1c_ reductions (− 1.70%) after 6 months, without significant differences between groups. In addition, after 6 months, in both groups the proportion of patients with HbA1c <7.0% increased from a small minority to almost one-third, while about 70% achieved levels <8%. Given the very high HbA_1c_ and FBG levels at initiation of 2BI, this result can be considered clinically meaningful.

In the subgroup of patients with HbA_1c_ values available at 12 months, HbA_1c_ reduction was sustained in the two groups and numerically greater with Gla-300.

After 6 months, FBG was reduced by about 60 mg/dl in both groups and only minor changes in body weight were recorded. Insulin dose up titration was modest (+4 U/day) but statistically significant; during the first 6 months of treatment, 0.2 U/kg of basal insulin was used in both groups.

Concerning safety, we found a similar profile of the two 2BIs. Indeed, incidence of hypoglycemic episodes ≤70 mg/dl and <54 mg/dl during 6 months was very low and similar in the two groups, and no severe hypoglycemic episodes were recorded on EMRs.

This study adds important insights into the understanding of clinical profile of T2D patients initiating Gla-300 or Deg-100 in the real world. Before PSM, socio-demographic and clinical characteristics of patients initiating Gla-300 or Deg-100 were similar. Only small between-group differences emerged in the concomitant use of some classes of diabetes therapies (secretagogues, glitazones and SGLT2i); these differences are likely to reflect the evolving scenario of T2D pharmacotherapy when the two 2BI were made available in Italy.

Our results underline the effectiveness and safety of both 2BI. Furthermore, the very high baseline HbA_1c_ (>9.0%) and FBG (>200 mg/dl) levels at the time of insulin initiation highlight once more the well-known problem of clinical inertia [3], suggesting a late treatment intensification. After 6 months, despite relevant improvements, many patients were still above the recommended HbA_1c_ and FBG targets in both groups. Low doses of basal insulin were adopted at 6-month follow-up of this real-world context (0.2 U/Kg) as compared to RCTs (0.3–0.8 U/Kg) [11–19], also considering that 0.2 U/kg should be the starting dose according to the Gla-300 label. However, a titration beyond 6 months from starting insulin therapy cannot be excluded in a real-life setting.

Results of our study were comparable to those of RCTs including insulin-naïve T2D cohorts treated with Gla-300 or Deg-100. The EDITION 3 study [13] showed that in the Gla-300 group, HbA_1c_ at T0 was of 8.51%, and at T6, it was reduced by 1.42%; the proportion of patients reaching HbA1c <7.0 at T6 was 43.1%. In the BEGIN Once-long [18], in the arm treated with Deg-100, HbA_1c_ decreased from 8.2% at T0 by 1.06% after 6 months. In the first head-to-head RCT (BRIGHT) [19], HbA_1c_ improved similarly in the two groups from 8.6 to 8.7% to 7.0% after 6 months. In the same study, comparable rates of hypoglycemia in the Gla-300 vs. Deg-100 group were documented (9.3 and 10.8 events per patient-year for hypoglycemia <=70 mg/dl and 0.6 versus 0.9 events per patient-year for hypoglycemia <54 mg/dl). A weight gain of about 2 Kg was documented in both groups.

Results of our study are also comparable with those obtained in the US real-world setting among insulin-naïve patients with T2D [23]. RESTORE-2 study shows very similar results to DELIVER D naïve study [23], where HbA_1c_ reduction was comparable in the Gla-300 and Deg-100 cohorts (− 1.67% vs. − 1.58%; *p* = 0.51), as was HbA_1c_ target attainment (HbA_1c_ <7%: 23.8% and 27.4%; *p* = 0.20; HbA1c <8%: 55.0% and 57.1%; *p* = 0.63). Furthermore, similarly low rates of hypoglycemia were reported in the two groups. In the LIGHTING study [24], data were collected from the Optum Humedica US electronic health records database. In the naïve matched cohort, a HbA_1c_ reduction of 1.5% was detected in patients treated with Gla-300 and Deg-100. Furthermore, severe hypoglycemic event rates with Gla-300 (estimated though a predictive modeling approach) were not different from those with Deg-100, whereas predicted rates of non-severe hypoglycemia were significantly lower with Gla-300 versus all comparators (first- and second-generation basal insulins). In the CONFIRM study [22], significantly greater HbA_1c_ reduction, larger reductions in hypoglycemia rates and lower risk of treatment discontinuation were demonstrated with Deg-100 versus Gla-300. However, these results should be interpreted with caution for at least two reasons: i) Both HbA_1c_ and hypoglycemia were analyzed in subgroups of the PS matched cohort without any further check on between-group imbalance and ii) pattern of baseline medications and an initial daily insulin dose of 40 U are poorly consistent with the expected profile of insulin-naïve patients [23]. Furthermore, there was no match with regard to hypoglycemia rates prior to insulin initiation and there was an imbalance that could per se drive the differences of the changes in hypoglycemia rates with Deg-100 versus Glar-300 [26].

A study on T1D (RESTORE-1) based on the same methodology described here has recently been published [27]. In agreement with RESTORE-1 findings, the take-home message from our new RESTORE-2 is the confirmation of a similar effectiveness and safety of the two 2BI. Yet, the need to overcome clinical inertia is still not met. Late insulin initiation and slow titration are likely due at least in part to the fear of hypoglycemia and weight gain. From a methodological standpoint, both studies confirm the importance of the secondary use of preexisting data for clinical research purposes. In this respect, the Italian network of diabetes centers adopting the same EMRs system represents a unique opportunity to conduct large, real-world effectiveness studies.

Among notable strengths of this study, we underline that this is the first RWE comparative study conducted outside the USA on the effectiveness and safety of 2BIs in T2D. Thanks to the large sample of patients with T2D routinely cared for by centers located in different areas of Italy, and the efficient use of EMR for research purposes, our data have good generalizability to the population of individuals with T2D followed under diabetologist care in Italy. The comparison of hypoglycemic episodes was based on documented events, with the caveat that only a subset of patients had their SMBG downloaded on EMRs. Indeed, the main limitation of this retrospective analysis was the lack of information on SMBG tests for a relevant proportion of patients, although the performed analysis is robust due to the large number of SMBG tests analyzed, the post-matching balance between the two groups, and the lack of difference in baseline risk of hypoglycemia. The download of SMBG values from glucose meters on EMRs was not a common practice in participating centers, suggesting the need to implement the systematic revision of SMBG data through EMRs.

Furthermore, between-group comparison was at least partially limited by the modest dose titration, preventing a head-to-head comparison between the two insulins when optimally used. Nevertheless, data reflect real-life practice and the barriers to optimize insulin doses, especially when patients or diabetologists are concerned by the risk of hypoglycemia. This is a key point deserving consideration in view of the importance of bringing more patients to their HbA_1c_ target.

In conclusion, in this comparative real-world study with PS matched cohorts of adult patients with T2D, initiating Gla-300 or Deg-100 was associated with similar improvements in glycemic control both in medium and long term, without weight gain and with low rate of hypoglycemia, with no severe episodes observed during an average of 6-month follow-up. The RESTORE-2 study confirms the effectiveness and safety of Gla-300, with comparable results with respect to Deg-100.

## Data Availability

The datasets generated during and/or analyzed during the current study are available from the corresponding author on reasonable request. Qualified researchers may request access to patient-level data and related documents [including, e.g., the clinical study report, study protocol with any amendments, blank case report form, statistical analysis plan and dataset specifications]. Patient-level data will be anonymized, and study documents will be redacted to protect the privacy of trial participants.

## Appendix 1: Variables used in PS matching—pre- and post-PSM baseline patients’ characteristics (ITT population)

**Table:**

Variable	Category	PRE-PSM				POST-PSM			
		Gla-300	Deg-100	*p*-value	Standardized difference	Gla-300	Deg-100	*p*-value	Standardized difference
N group		808	358		.	357	357		.
BMI (Kg/m^2^)		29.31 (5.79)	29.89 (5.64)	0.07	10.0026	29.91 (6.05)	29.86 (5.62)	0.75	− 0.83529
GLP1-RAs (%)	No	658 (81.44)	274 (76.54)	0.054	− 12.0474	278 (77.87)	273 (76.47)	0.66	− 3.33727
	Yes	150 (18.56)	84 (23.46)		.	79 (22.13)	84 (23.53)		.
SGLT2 inhibitors (%)	No	586 (72.52)	288 (80.45)	0.004	18.7626	290 (81.23)	287 (80.39)	0.78	− 2.13416
	Yes	222 (27.48)	70 (19.55)		.	67 (18.77)	70 (19.61)		.
Glitazones (%)	No	760 (94.06)	318 (88.83)	0.002	− 18.7885	325 (91.04)	318 (89.08)	0.38	− 6.55581
	Yes	48 (5.94)	40 (11.17)		.	32 (8.96)	39 (10.92)		.
Secretagogues (%)	No	464 (57.43)	179 (50.00)	0.02	− 14.9341	196 (54.90)	179 (50.14)	0.20	− 9.5468
	Yes	344 (42.57)	179 (50.00)		.	161 (45.10)	178 (49.86)		.

Data are means and standard deviations or frequencies and proportions.

*p*-values derived from unpaired t-test or the Mann–Whitney U-test in case of continuous variables and the chi-square test or Fisher exact test for categorical variables, as appropriate. Statistically significant p-values (*p*<0.05) are in bold. A standardized mean difference less than 10 (absolute values) indicates a good balance between groups.

## Appendix 3: Safety population—baseline patients’ characteristics—pre- and post-PSM

**Table:**

Variable	PRE-PSM			POST-PSM		
	Gla-300	Deg-100	*p*-value	Gla-300	Deg-100	*p*-value
N group	241	129		123	123	
Age (years)	70.2 (11.0)	69.6 (10.5)	0.48	70.0 (11.0)	69.7 (10.4)	0.71
Males (%)	144 (59.8)	78 (60.5)	0.89	78 (63.4)	76 (61.8)	0.79
Diabetes duration (years)	14.21 (10.7)	13.12 (7.8)	0.39	13.0 (8.5)	13.2 (7.9)	0.70
BMI (Kg/m^2^)	29.3 (6.0)	29.1 (5.0)	0.82	29.5 (6.2)	29.2 (5.0)	1.00
HbA1c (%)	8.6 (1.4)	8.7 (1.5)	0.92	8.7 (1.5)	8.7 (1.4)	0.93
HbA1c (mmol/l)	70.5 (15.4)	71.6 (15.9)	0.94	71.4 (16.1)	70.9 (14.9)	0.87
Fasting blood glucose (mg/dl)	182.1 (55.4)	191.3 (58.1)	0.21	190.5 (60.9)	188.2 (57.5)	0.80
Systolic blood pressure (mmHg)	135.2 (19.7)	133.6 (15.9)	0.43	133.3 (18.0)	133.7 (15.9)	0.71
Diastolic blood pressure (mmHg)	75.9 (10.0)	77.6 (10.4)	0.46	74.7 (10.2)	77.6 (10.2)	0.07
Total cholesterol (mg/dl)	171.7 (40.7)	181.1 (43.6)	0.10	175.1 (44.6)	179.8 (42.8)	0.32
LDL-cholesterol (mg/dl)	91.7 (33.5)	101.6 (39.5)	0.06	94.8 (36.6)	100.9 (39.0)	0.34
HDL-cholesterol (mg/dl)	47.4 (13.5)	45.4 (11.0)	0.36	46.7 (12.6)	45.4 (10.4)	0.59
Triglycerides (mg/dl)	157.2 (86.7)	174.7 (156.3)	0.77	154.8 (87.5)	171.0 (158.4)	0.84
eGFR <60 ml/min*1.73m^2^	45 (41.3)	13 (41.9)	0.95	18 (35.3)	10 (37.0)	0.89
Microalbuminuria (%)	34 (31.78)	18 (29.51)	0.76	18 (35.29)	17 (29.31)	0.50
Diabetes complications (%)	17 (7.1)	7 (5.4)	0.54	6 (4.9)	7 (5.7)	0.78
*Glucose-lowering therapy*						
Daily basal insulin dose (U)	12.2 (5.9)	12.6 (7.2)	0.69	12.6 (6.3)	12.4 (6.9)	0.23
*No. of glucose-lowering drugs other than insulin*						
<2	32 (13.3)	24 (18.6)	0.17	18 (14.6)	20 (16.3)	0.72
>=2	209 (86.7)	105 (81.4)		105 (85.4)	103 (83.7)	
Metformin (%)	202 (83.8)	100 (77.5)	0.13	102 (82.9)	97 (78.9)	0.42
Secretagogues (%)	92 (38.2)	60 (46.5)	0.12	56 (45.5)	57 (46.3)	0.90
Glitazones (%)	20 (8.3)	12 (9.3)	0.74	9 (7.3)	12 (9.8)	0.49
Acarbose (%)	18 (7.5)	8 (6.2)	0.65	5 (4.1)	8 (6.5)	0.39
DPPIV inhibitors (%)	129 (53.5)	63 (48.8)	0.39	66 (53.7)	62 (50.4)	0.61
GLP1-RAs (%)	60 (24.9)	37 (28.7)	0.43	25 (20.3)	36 (29.3)	0.10
SGLT2 inhibitors (%)	80 (33.2)	21 (16.3)	**0.0005**	31 (25.2)	21 (17.1)	0.12
Mean no. of available SMBG tests per patient in the study period (%)	178.8 (446.5)	158.4 (163.8)	0.47	149.2 (108.2)	159.5 (167.4)	0.92

Data are means and standard deviations or frequencies and proportions.

Variables included in the PSM: No. of glucose-lowering drugs other than insulin, diabetes duration, use of metformin and secretagogues, HbA1c.

*p*-values derived from unpaired t-test or the Mann-Whitney U-test in case of continuous variables and the chi-square test or two-sided Fisher exact test for categorical variables, as appropriate. Statistically significant p-values (*p*<0.05) are in bold.

## Appendix 4: Safety population: variables used in PS matching—pre- and post-PSM baseline patient characteristics

**Table:**

Variable	Category	PRE-PSM				POST-PSM			
		Gla-300	Deg-100	*p*-value	Standardized difference	Gla-300	Deg-100	p-value	Standardized difference
*N* group		241	129		.	123	123		.
No. of glucose-lowering drugs other than insulin (%)	<2	32 (13.28)	24 (18.60)	0.1731	14.5899	18 (14.63)	20 (16.26)	0.7242	4.5004
	>=2	209 (86.72)	105 (81.40)		.	105 (85.37)	103 (83.74)		.
Diabetes duration in classes (%)	<=5 years	37 (15.35)	17 (13.18)	0.1355	− 6.2205	18 (14.63)	17 (13.82)	0.8504	− 2.3275
	6-10 years	44 (18.26)	29 (22.48)		.	34 (27.64)	27 (21.95)		.
	11-20 years	111 (46.06)	53 (41.09)		.	46 (37.40)	52 (42.28)		.
	>20 years	46 (19.09)	23 (17.83)		.	22 (17.89)	23 (18.70)		.
	NA	3 (1.24)	7 (5.43)		.	3 (2.44)	4 (3.25)		.
Metformin (%)	No	39 (16.18)	29 (22.48)	0.1361	15.9995	21 (17.07)	26 (21.14)	0.4174	10.3539
	Yes	202 (83.82)	100 (77.52)		.	102 (82.93)	97 (78.86)		.
Secretagogues (%)	No	149 (61.83)	69 (53.49)	0.1203	-16.9341	67 (54.47)	66 (53.66)	0.8982	− 1.6315
	Yes	92 (38.17)	60 (46.51)		.	56 (45.53)	57 (46.34)		.
HbA1c in classes	3.0-6.9%	23 (9.54)	5 (3.88)	0.0095	− 22.7998	4 (3.25)	5 (4.07)	0.9891	4.3315
	7.0-8.0%	63 (26.14)	50 (38.76)		.	48 (39.02)	47 (38.21)		.
	8.1-9.0%	90 (37.34)	34 (26.36)		.	34 (27.64)	34 (27.64)		.
	>9.0%	65 (26.97)	40 (31.01)		.	37 (30.08)	37 (30.08)		.

Data are means and standard deviations or frequencies and proportions.

p-values derived from unpaired t-test or the Mann–Whitney U-test in case of continuous variables and the chi-square test or Fisher exact test for categorical variables, as appropriate. Statistically significant p-values (*p*<0.05) are in bold. A standardized mean difference less than 10 (absolute values) indicates a good balance between groups.

## Appendix 5: Changes in estimated mean levels of HbA1c in the subgroup of ITT PS-matched naive population with HbA1c values at T12 (*N*=121 in Gla-300 group and *N*=133 in Deg-100 group)

**Table:**

Visit	Gla-300			Deg-100			
	Estimated mean and 95% CI	Estimated mean difference from T0 and 95% CI	Within group *p*-value*	Estimated mean and 95% CI	Estimated mean difference from T0 and 95% CI	Within group *p*-value*	Between-group *p*-value**
T0	9.25 (9.07;9.43)	–	–	9.18 (9.00; 9.36)	–	–	
T12	7.55 (7.37;7.73)	− 1.71 (− 1.94; − 1.48)	**<0.0001**	7.74 (7.56;7.92)	− 1.44 (− 1.67; − 1.21)	**<0.0001**	0.052

* Estimates and Paired t-test derived from linear mixed models for repeated measurements.

**One side Paired t-test derived from linear mixed models for repeated measurements.

Statistically significant *p*-values (*p*<0.05) are in bold.

## Appendix 6: Incidence rate of hypoglycemic events ≤70 mg/dl and <54 mg/dl by treatment at T0 (Safety Naïve population: PS matched patients having at least 1 SMBG value available)

**Table:**

Endpoint	Group	Subjects	No. SMBG tests	Events	Person-months	IR (95%CIs)	IRR (95% CIs)	Between-group *p*-value
BG ≤70 mg/dl	Gla-300	123	4,781	14	274.7	0.05 (0.02;0.14)	0.55 (0.16;1.88)	0.34
	Deg-100	123	6,367	24	259	0.09 (0.04;0.20)	–	–
BG <54 mg/dl	Gla-300	123	4,781	4	274.7	0.01 (0.00;0.06)	0.42 (0.07;2.36)	0.32
	Deg-100	123	6,367	9	259	0.03 (0.01;0.09)	–	.

*p*-values derived from Poisson regression models with correction for overdispersion. IR=incidence rate (number of events per person-months); IRR=incidence rate ratio; 95%CI=95% Confidence Intervals

## Abbreviations

ADA: American diabetes association
BMI: Body mass index
Deg-100: Degludec 100 U/ml
EMR: Electronic medical record
FB: Fasting Blood Glucose
Gla-300: Glargine 300 U/ml
HbA1c: Glycated hemoglobin
IR: Incidence rates
PS: Propensity score
PSM: Propensity score-matched
RCT: Randomized clinical trials
2BI: Second-generation basal insulin analogues
SMBG: Self-monitoring blood glucose tests
T1D: Type 1 diabetes
T2D: Type 2 diabetes
US: United States

## References

[1] Buse JB, Wexler DJ, Tsapas A (2020). update to: Management of hyperglycaemia in type 2 diabetes, 2018. A consensus report by the American Diabetes Association (ADA) and the European Association for the Study of Diabetes (EASD). Diabetologia.

[2] AMD/SID. Standard italiani per la cura del diabete mellito 2018. https://www.siditalia.it/pdf/Standard%20di%20Cura%20AMD%20-%20SID%202018_protetto2.pdf [last access 29 June 2021]

[3] Khunti K, Gomes MB, Pocock S (2018). Therapeutic inertia in the treatment of hyperglycaemia in patients with type 2 diabetes: a systematic review. Diabetes Obes Metab.

[4] Andreozzi F, Candido R, Corrao S (2020). Clinical inertia is the enemy of therapeutic success in the management of diabetes and its complications: a narrative literature review. Diabetol Metab Syndr.

[5] Ceriello A, deValk HW, Guerci B (2020). The burden of type 2 diabetes in Europe: current and future aspects of insulin treatment from patient and healthcare spending perspectives. Diabetes Res Clin Pract.

[6] Madenidou AV, Paschos P, Karagiannis T (2018). Comparative benefits and harms of basal insulin analogues for Type 2 diabetes: a systematic review and network meta-analysis. Ann Intern Med.

[7] Becker RH, Dahmen R, Bergmann K, Lehmann A, Jax T, Heise T (2015). New insulin glargine 300 Units mL-1 provides a more even activity profile and prolonged glycemic control at steady state compared with insulin glargine 100 Units mL-1. Diabetes Care.

[8] Becker RHA, Nowotny I, Teichert L, Bergmann K, Kapitza C (2015). Low within- and between-day variability in exposure to new insulin glargine 300 U/ml. Diabetes Obes Metab.

[9] Heise T, Hermanski L, Nosek L (2012). Insulin degludec: four times lower pharmacodynamic variability than insulin glargine under steady-state conditions in type 1 diabetes. Diabetes Obes Metab.

[10] Goldman J, Kapitza C, Pettus J, Heise T (2017). Understanding how pharmacokinetic and pharmacodynamic differences of basal analog insulins influence clinical practice. Curr Med Res Opin.

[11] Bolli GB, Ziemen M, Muehlen-Bartmer I, Bizet F, Home PD (2014). New insulin glargine 300 units/mL versus glargine 100 units/ml in people with type 2 diabetes using basal and mealtime insulin: glucose control and hypoglycemia in a 6-month randomized controlled trial (Edition I). Diabetes Care.

[12] Yki-Järvinen H, Bergenstal R, Ziemen M (2014). New insulin glargine 300 units/mL versus glargine 100 units/mL in people with type 2 diabetes using oral agents and basal insulin: glucose control and hypoglycemia in a 6-month randomized controlled trial (Edition 2). Diabetes Care.

[13] Bolli GB, Riddle MC, Bergenstal RM (2015). New insulin glargine 300 U/mL compared with glargine 100 U/mL in insulin-naïve people with type 2 diabetes on oral glucose-lowering drugs: a randomized controlled trial (Edition 3). Diabetes Obes Metab.

[14] Home PD, Bergenstal RM, Bolli GB (2015). New insulin glargine 300 units/mL versus glargine 100 units/mL in people with type 1 diabetes: a randomized, phase 3a, open-label clinical trial (EDITION 4). Diabetes Care.

[15] Meneghini L, Atkin SL, Gough SC (2013). The efficacy and safety of insulin degludec given in variable once-daily dosing intervals comparedwith insulin glargine and insulin degludec dosed at the same time daily. Diabetes Care.

[16] Mathieu C, Hollander P, Miranda-Palma B (2013). Efficacy and safety of insulin degludec in a flexible dosing regimen vs insulin glargine in patients with type 1 diabetes (BEGIN: Flex T1): a 26-week randomized, treat-to-target trial with a 26-week extension. J Clin Endocrinol Metab.

[17] Heller S, Buse J, Fisher M (2012). Insulin degludec, an ultra-longacting basal insulin, versus insulin glargine in basal-bolus treatment with mealtime insulin aspart in type 1 diabetes (BEGIN Basal-Bolus Type 1): a phase 3, randomised, open-label, treat-to-target non-inferiority trial. Lancet.

[18] Zinman B, Philis-Tsimikas A, Cariou B (2012). Insulin degludec versus insulin glargine in insulin-naive patients with type 2 diabetes: a 1-year, randomized, treat-to-target trial (BEGIN Once Long). Diabetes Care.

[19] Rosenstock J, Cheng A, Ritzel R (2018). More similarities than differences testing insulin glargine 300 units/mL versus insulin degludec 100 units/mL in insulin-naive type 2 diabetes: the randomized head-to-head BRIGHT trial. Diabetes Care.

[20] Philis-Tsimikas A, Klonoff DC, Khunti K (2020). Risk of hypoglycaemia with insulin degludec versus insulin glargine U300 in insulin-treated patients with type 2 diabetes: the randomised, head-to-head CONCLUDE trial. Diabetologia.

[21] Zaccardi F, Davies MJ, Khunti K (2020). The present and future scope of real-world evidence research in diabetes: what questions can and cannot be answered and what might be possible in the future?. Diabetes Obes Metab.

[22] Tibaldi J, Hadley-Brown M, Liebl A (2019). A comparative effectiveness study of degludec and insulin glargine 300 U/mL in insulin-naïve patients with type 2 diabetes. Diabetes Obes Metab.

[23] Sullivan SD, Nicholls CJ, Gupta RA (2019). Comparable glycaemic control and hypoglycaemia in adults with type 2 diabetes after initiating insulin glargine 300 units/mL or insulin degludec: the DELIVER Naïve D real-world study. Diabetes Obes Metab.

[24] Pettus J, Roussel R, Liz Zhou F (2019). Rates of hypoglycemia predicted in patients with type 2 diabetes on insulin glargine 300 U/ml versus first- and second-generation basal insulin analogs: the real-world LIGHTNING study. Diabetes Ther.

[25] Austin PC (2009). Balance diagnostics for comparing the distribution of baseline covariates between treatment groups in propensity-score matched samples. Stat Med.

[26] Freemantle N, Jourdan S (2019). Comment on "a comparative effectiveness study of degludec and insulin glargine 300 U/mL in insulin-naïve patients with type 2 diabetes". Diabetes Obes Metab.

[27] Laviola L, Porcellati F, Bruttomesso D (2021). Comparative effectiveness of switching from first-generation basal insulin to glargine 300 U/ml or degludec 100 U/ml in type 1 diabetes: the RESTORE-1 study. Diabetes Ther.

